# Evaluation of emergency call Code Blue over a 5-year period

**DOI:** 10.1186/cc14491

**Published:** 2015-03-16

**Authors:** N Bakan, G Karaoren, S Tomrk, S Keskin

**Affiliations:** 1 Istanbul Umraniye Training and Research Hospital, Istanbul, Turkey

## Introduction

Code systems are the emergency call and management systems for rapid response in healthcare institutions. The main aim of these systems is to provide common institutional understanding of what is necessary to be done immediately at the time of an event. Code Blue (CB), which is used throughout the world and was described in the 2008 service quality standards of Turkey, defines the necessary emergency intervention in cases of respiratory or cardiac arrest. This study aimed to evaluate the clinical and application data of patients for whom a CB call was made between 2009 and 2013.

## Methods

After approval of local ethics committee, retrospective examination was made of CB forms. The age and gender of the patient, diagnosis, department to which admitted, time of CB call, reason for CB, whether or not CB was appropriate, whether or not CPR was applied, duration of CPR if applied, APACHE II and PRISM scores and predicted mortality were recorded from the hospital automated record system and the CB form. Patients who refused treatment or who could not reach the necessary parameters for the calculation of APACHE II and PRISM scores were excluded.

## Results

From CB calls for a total of 1,195 patients over the 5-year period, 1,035 (86.6%) were evaluated. The rate of erroneous CB was 36.9%. Patients comprised 413 (39.9%) females and 622 (60.1%) males with a mean age of 59.73 ± 23.13 years (range, 0.1 to 102 years). The distribution of total cases over the 5 years (2009 to 2013) was 15.5%, 25.2%, 26.5%, 19% and 13.8% respectively. Distribution according to clinic was emergency internal (37.5%), internal (16.5%) and emergency surgical (9.5%). Clinical diagnosis was cardiac 28.8%, neurological 15.6% and end-stage cancer 13.5%. A total 19.9% of the patients were those discharged from intensive care. The total survival rate was 59.6%. The duration of CPR in survivors was statistically longer than in nonsurvivors (*P *< 0.01). There was no statistically significant relationship between the duration of CPR and age (*P >*0.05). The overall mean time taken to reach the patient was 102.71 ± 22.47 seconds, which reduced to 93.64 ± 19.91 seconds in 2013. The APACHE II-PRISM scores and mortality rates were low in the cases of erroneous CB (*P *< 0.05).

## Conclusion

The time taken to reach patients conformed with the global standard mean 2 to 3 minutes [1]. The rates of erroneous CB and time to reach patients reduced each year due to more staff experienced and knowledgeable in CB and the structuring of the emergency clinic.

## References

[B1] Nolan JP European resuscitation council guidelines for resuscitation 2010 section 1. Executive summary Resuscitation 2010 81 1219 76 20956052 10.1016/j.resuscitation.2010.08.021

